# Reframing brain aging: neuroinflammation as an interconnected network process

**DOI:** 10.3389/fragi.2026.1858763

**Published:** 2026-07-15

**Authors:** Ludmila Müller, Svetlana Di Benedetto, Viktor Müller

**Affiliations:** Max Planck Institute for Human Development, Center for Lifespan Psychology, Berlin, Germany

**Keywords:** blood-brain barrier, brain aging, glial activation, gut-brain axis, neuroimmune and neuroinflammatory networks, neuroinflammation, neurovascular interface, systems approach

## Abstract

Neuroinflammation has emerged as a central component of brain aging, shaping the balance between neural resilience and vulnerability to cognitive decline. Rather than representing a simple consequence of neuronal damage, neuroinflammatory processes are increasingly recognized as active regulators of synaptic integrity, neuronal survival, and circuit function. Under physiological conditions, neuroimmune signaling may contribute to tissue homeostasis, synaptic maintenance, and adaptive responses to cellular stress; however, persistent or dysregulated inflammatory activity may disrupt these functions, promoting network instability and increasing vulnerability to age-related pathology. These processes arise from complex interactions among neurons, glial cells, vascular elements, and peripheral immune signals that together form dynamic neuroimmune networks. Within the aging brain, microglia and astrocytes play key roles in coordinating immune surveillance, synaptic remodeling, and inflammatory signaling. Age-related alterations in glial function can disrupt homeostatic communication within neuron–glia networks, promoting persistent low-grade inflammation and altered synaptic regulation. Importantly, neuroinflammatory activity in the brain is strongly influenced by systemic factors, including peripheral immune aging, changes in blood–brain barrier integrity, and signals originating from the gut–brain axis. In this mini-review, we discuss brain aging from a network perspective, emphasizing how multiscale interactions between cellular and systemic processes shape neuroinflammatory trajectories across the lifespan. We further highlight emerging approaches—including multi-omics technologies, advanced neuroimaging, and systems-level analyses—that are enabling a more integrated understanding of neuroinflammatory dynamics. Viewing neuroinflammation as a network phenomenon may provide new insights into mechanisms of cognitive aging and identify potential targets for strategies aimed at preserving brain health.

## 1 Introduction: brain aging as a neuroimmune network process

The aging brain undergoes a wide range of structural, cellular, and functional alterations that can influence cognitive performance and susceptibility to neurological disease (Zou et al., 2025; Zhang R. et al., 2025; Shafqat et al., 2023; Di Benedetto et al., 2017; Kempuraj et al., 2026). Among the processes increasingly associated with brain aging, neuroinflammation has attracted considerable attention as a potential modulator of neural function and resilience. Accumulating evidence suggests that neuroinflammatory processes may actively participate in shaping aging trajectories within the central nervous system (CNS), including mechanisms involved in synaptic regulation, neural circuit stability, and brain resilience. However, the mechanisms through which inflammatory signaling influences brain aging remain incompletely understood and may depend on complex interactions between cellular, systemic, and environmental factors (Müller et al., 2025a; Ransohoff, 2016; Müller et al., 2025b).

Within the CNS, immune-related processes are largely mediated by glial cells, particularly microglia and astrocytes, which contribute to immune surveillance, synaptic remodeling, and tissue homeostasis (Colonna and Butovsky, 2017; Kierdorf and Prinz, 2017; Müller and Di Benedetto, 2025a). Experimental studies *in vitro* and in animal models indicate that microglia can respond dynamically to changes in the neural environment and may regulate synaptic turnover, neuronal connectivity, and inflammatory signaling pathways. Astrocytes may further modulate these processes through the release of cytokines, chemokines, and metabolic factors that influence neuronal activity and glial communication (Linnerbauer et al., 2020; Pacca-Corrêa et al., 2026; Beard et al., 2021; Singh, 2022).

With advancing age, both microglial and astrocytic functions can change, potentially leading to altered inflammatory signaling (Müller et al., 2025a; Müller and Di Benedetto, 2025a). Evidence from animal studies suggests that aging microglia may exhibit features often described as “priming,” characterized by heightened responsiveness to inflammatory stimuli, whereas astrocytes may display altered reactive states. Observations from human post-mortem studies and neuroimaging investigations also suggest that age-related changes in glial activity can occur in the human brain, although the precise functional consequences remain under active investigation (Norden et al., 2015; Perry and Holmes, 2014; Henstridge et al., 2015; La Sala and Farini, 2025).

Additional CNS-associated immune populations located at CNS interfaces, including border-associated macrophages and meningeal immune cells, are increasingly recognized as integral components of neuroimmune regulation and CNS homeostasis during aging. Their roles and underlying mechanisms have been extensively characterized in the literature and are discussed in detail in several excellent reviews. Accordingly, they are only briefly addressed in the present mini-review (Kierdorf et al., 2019; Smyth and Kipnis, 2025; Prinz et al., 2021).

Importantly, neuroinflammation in aging may not arise solely from processes intrinsic to the brain. Increasing evidence indicates that systemic physiological changes accompanying aging can influence neuroimmune signaling. For example, alterations in peripheral immune function—referred to as immunosenescence—may lead to changes in circulating inflammatory mediators that could affect CNS function (Kempuraj et al., 2026; Müller and Di Benedetto, 2025a; Müller and Di Benedetto, 2024; Müller and Di Benedetto, 2026; Müller and Di Benedetto, 2025b). Experimental studies in animal models suggest that age-related changes in blood–brain barrier (BBB) integrity may permit greater exchange of immune signals between the peripheral circulation and the brain. In humans, imaging and biomarker studies have reported associations between systemic inflammatory markers and measures of cognitive function or brain structure, although causal relationships remain difficult to establish (Knox et al., 2022; Gonzalez-Bautista et al., 2025; Mar et al., 2015).

In addition, emerging research suggests that interactions between the CNS and other physiological systems, including the gut microbiome and metabolic networks, may contribute to neuroinflammatory processes (Yu et al., 2022; Kohler et al., 2016; Strasser and Ticinesi, 2023; Müller and Di Benedetto, 2025c). Studies in animal models indicate that microbiome composition can influence microglial maturation and immune signaling in the brain, while early human studies suggest potential links between microbial metabolites, systemic inflammation, and cognitive health during aging (Müller and Di Benedetto, 2025c; Erny et al., 2015; Bagheri et al., 2026). These findings support the view that neuroinflammation may arise from interconnected networks spanning cellular, tissue, and systemic levels.

Taken together, available evidence suggests that neuroinflammation may represent a dynamic and context-dependent process in brain aging, reflecting interactions between neural, glial, vascular, and systemic factors. In this mini-review, we consider neuroinflammation from a network perspective, emphasizing how cellular neuroimmune circuits interact with systemic physiological processes to shape aging trajectories of the brain. We discuss current insights into cellular neuroimmune interactions, systemic modulators of brain inflammation, and the potential implications of these networks for cognitive aging and vulnerability to neurodegenerative disease.

## 2 Cellular neuroimmune networks in the aging brain

**FIGURE 1:**
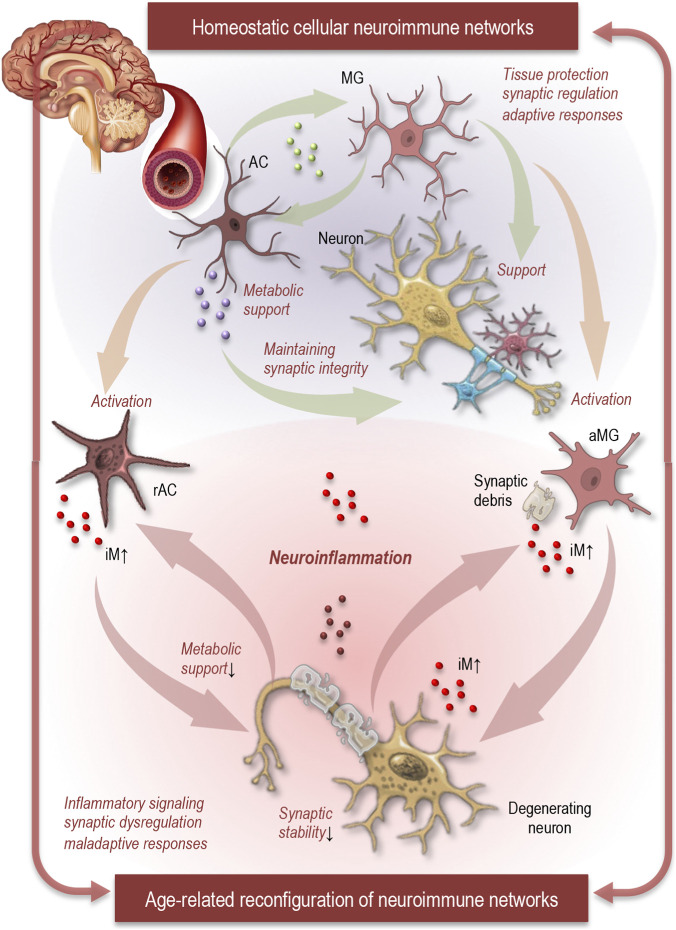
Cellular neuroimmune networks in the aging brain. A simplified schematic illustrates interactions among neurons, microglia (MG), and astrocytes (AC) that form cellular neuroimmune networks within the CNS. The upper blue region represents homeostatic conditions, in which coordinated communication between neurons and glial cells supports neuronal function and synaptic stability. Microglia act as central regulators of local immune signaling by surveying the neural environment and interacting with neurons through signaling pathways that contribute to synaptic maintenance and tissue homeostasis. Astrocytes serve as coordinators of neuronal and immune signals by regulating metabolic support, extracellular signaling, and neuron–glia communication. Together, these interactions contribute to balanced neuroimmune regulation within neural circuits. The lower red region represents age-associated neuroinflammatory conditions, in which the dynamics of neuron–glia communication may shift toward enhanced inflammatory signaling. Microglia may adopt activated states (aMG) associated with increased release of inflammatory mediators (iM), while astrocytes can transition to reactive phenotypes (rAC) that further modify the local signaling environment. These changes may influence neuron–glia communication and synaptic regulation, potentially affecting synaptic remodeling and neuronal integrity. The schematic highlights how alterations in glial activity may shift cellular neuroimmune networks from homeostatic regulation toward inflammatory states that could contribute to increased vulnerability of neuronal circuits during brain aging.

Cellular interactions within the CNS form highly coordinated neuroimmune networks that contribute to tissue protection, synaptic regulation, and adaptive responses to physiological challenges. These networks involve continuous communication between neurons and multiple glial cell types, mediated through direct contact, soluble signaling molecules, and metabolic coupling (Figure 1). Aging may alter the dynamics of these interactions, potentially modifying how inflammatory signals are generated, transmitted, and resolved within neural circuits (Müller et al., 2025a; Müller et al., 2025c).

### 2.1 Microglia as central regulators of neuroimmune signaling

Microglia occupy a central position within cellular neuroimmune networks (Figure 1) due to their capacity to integrate signals from neurons, glia, and the extracellular environment. Microglial inflammatory signaling may influence synaptic remodeling and neuronal excitability, while neuronal stress-associated signals can in turn further modulate microglial activation states (Müller and Di Benedetto, 2025a; Müller et al., 2025d). Experimental studies in cell culture systems and animal models suggest that microglia can respond to neurotransmitters, damage-associated molecules, and metabolic cues, thereby adjusting immune-related signaling within local brain regions. Through the release of cytokines, chemokines, and growth factors, microglia may influence neuronal activity, synaptic organization, and interactions with other glial populations (Nimmerjahn et al., 2005; Haynes et al., 2006; Domercq et al., 2013).

Age-related shifts in microglial signaling profiles have been reported in several animal studies, including transcriptomic and proteomic analyses in aged rodents, which consistently show increased expression of genes associated with inflammatory reactivity, altered phagocytic function, and changes in homeostatic markers. These alterations may reflect underlying disruptions in cellular energy metabolism, mitochondrial function, lipid handling, and stress-response pathways, as well as age-dependent remodeling of intracellular signaling cascades such as NF-κB, MAPK, and inflammasome-related pathways (Li et al., 2023; Kang et al., 2024; Pan et al., 2020). Together, these changes are supposed to modify how microglia detect, integrate, and respond to environmental cues, including neuronal activity, synaptic debris, and systemic inflammatory signals, thereby shifting their role in maintaining synaptic integrity and tissue homeostasis (Müller et al., 2025a).

In parallel, human neuroimaging studies using indirect markers of glial activity as well as advanced MRI-based measures sensitive to microstructural and metabolic changes—have reported age-associated differences in brain regions implicated in immune and metabolic regulation. These findings suggest that glial-related signals may increase or become more spatially heterogeneous with age, particularly in regions such as the hippocampus, basal ganglia, and cortical association areas (Pacca-Corrêa et al., 2026; Beard et al., 2021; Suridjan et al., 2014; Siegle et al., 2021; Wang et al., 2025). However, the cellular and molecular processes that give rise to these imaging signatures remain only partially resolved, as they likely reflect a composite of microglial, astrocytic, vascular, and systemic immune contributions. As such, linking *in vivo* imaging readouts to specific cellular states continues to be a key challenge in translating animal findings to the human brain. Collectively, the multiscale approaches may help distinguish physiological neuroimmune adaptations from dysregulated inflammatory states associated with neural vulnerability during aging.

### 2.2 Astrocytes as coordinators of neuronal and immune signals

Astrocytes contribute to neuroimmune communication by linking inflammatory signaling with metabolic and synaptic regulation (Figure 1). These cells form extensive contacts with neurons, blood vessels, and other glial cells, positioning them to coordinate local responses to physiological changes. Astrocytes may integrate neuronal activity-dependent signals and inflammatory cues, subsequently modulating synaptic homeostasis, metabolic support, and local inflammatory amplification within neural circuits. *In vitro* experiments and animal studies indicate that astrocytes can regulate extracellular neurotransmitter concentrations, provide metabolic substrates to neurons, and release signaling molecules that influence immune activity within the CNS (Müller et al., 2025a; Pacca-Corrêa et al., 2026; Lopez-Ortiz and Eyo, 2024; Lee et al., 2023).

During aging, astrocytic functions may shift in ways that alter their regulatory capacity, including changes in calcium signaling dynamics, neurotransmitter uptake efficiency, metabolic support of neurons, and maintenance of ion and water homeostasis. These alterations can affect the ability of astrocytes to buffer synaptic activity, regulate extracellular glutamate and potassium levels, and support energy coupling between blood vessels and neuronal networks (Müller et al., 2025d; Clarke et al., 2018; Pessoa et al., 2026). In addition, aging astrocytes may exhibit modified inflammatory responsiveness, with a tendency toward prolonged or dysregulated cytokine and chemokine release in response to stress signals. Such functional changes could ultimately reshape astrocyte–neuron and astrocyte–microglia communication, thereby influencing synaptic stability, network excitability, and the overall resilience of neural circuits during aging (Müller and Di Benedetto, 2025a; Müller et al., 2025c; Serrano et al., 2025).

Experimental observations in animal models suggest that astrocytes can display modified gene expression patterns and changes in signaling pathways related to inflammation, metabolism, and stress responses. Depending on context, such alterations could either support adaptive responses to cellular stress or contribute to prolonged inflammatory signaling. Evidence from human brain studies indicates that astrocyte-associated molecular signatures may change with age, although their functional significance *in vivo* remains to be fully clarified (Serrano et al., 2025; Linker et al., 2025; Krawczyk et al., 2022; Serrano-Pozo et al., 2024). Thus, age-associated shifts in astrocytic signaling may alter the balance between metabolic support and inflammatory amplification within local neuronal networks.

### 2.3 Neuron–glia communication and synaptic regulation

Communication between neurons and glial cells forms an essential component of neuroimmune networks in the brain (Figure 1). Neurons can release signaling molecules that influence microglial and astrocytic behavior, while glial cells can modulate neuronal activity through the secretion of cytokines, growth factors, and metabolic substrates (Müller and Di Benedetto, 2025a; Linnerbauer et al., 2020; Müller et al., 2025d; Song and Dityatev, 2018; Haidar et al., 2022). In experimental models, immune-related signaling pathways have been shown to participate in processes such as synaptic remodeling, elimination of damaged cellular components, and modulation of neuronal excitability. Molecular systems including cytokine networks, complement components, and purinergic signaling pathways may contribute to these forms of communication (Palasz et al., 2023; Charabati et al., 2023; Chavda et al., 2022; Hille et al., 2024; Guo et al., 2025; Mrza et al., 2024; Müller et al., 2026a).

Evidence from animal studies suggests that age-related alterations in these signaling pathways could affect synaptic maintenance and circuit organization. For example, dysregulation of immune-related synaptic remodeling mechanisms may influence the stability of neural connections under certain conditions (Balietti et al., 2012; Heuer et al., 2024; Radulescu et al., 2023). Changes in neuron–glia communication, particularly involving microglial and astrocytic sensing of synaptic activity and extracellular signaling molecules, may further contribute to shifts in synaptic strength and network connectivity over time (Müller et al., 2025a; Müller et al., 2025c; Müller et al., 2025d; Radulescu et al., 2023). However, direct evidence linking these mechanisms to functional outcomes in the human aging brain remains limited, and many proposed pathways are currently inferred from experimental models rather than directly observed *in vivo* in humans.

Importantly, the functional consequences of neuroimmune signaling may depend strongly on context, including signaling intensity, temporal dynamics, brain region, and cellular state. For example, complement-associated pathways that contribute to physiological synaptic remodeling under homeostatic conditions may also participate in excessive synaptic elimination during aging or neurodegenerative conditions (Lian et al., 2016; Chi et al., 2025; Stevens et al., 2007). Similarly, cytokine-mediated signaling can exert both adaptive and detrimental effects on neuronal plasticity depending on local microenvironmental conditions and the duration of inflammatory activation. Such context-dependent responses may contribute to the variability and occasional inconsistencies reported across experimental studies (Li and Flavell, 2008; Prieto and Cotman, 2017). Dysregulation of these signaling pathways could contribute to impaired synaptic plasticity and reduced circuit adaptability during aging.

### 2.4 Shifting dynamics within cellular neuroimmune networks

Collectively, these findings indicate that cellular neuroimmune networks may undergo gradual reconfiguration during aging. Alterations in signaling sensitivity, metabolic coupling, and intercellular communication could influence how neural circuits respond to physiological stressors or environmental challenges. Because these processes involve multiple interacting cell types, their functional consequences may depend on the broader cellular context and on systemic influences that shape inflammatory signaling within the brain. These observations suggest that aging-related alterations in neuron–glia communication may shift neuroimmune signaling from adaptive synaptic support toward persistent inflammatory activation, potentially affecting circuit stability and neuronal resilience.

## 3 Systemic integration of neuroinflammatory networks

**FIGURE 2:**
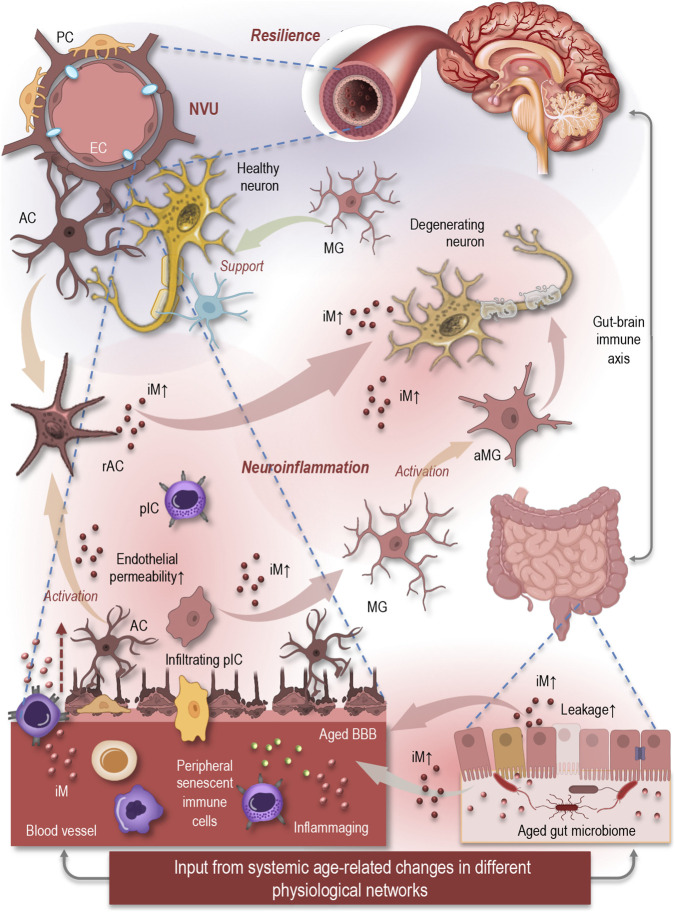
Systemic integration of neuroinflammatory networks during brain aging. A simplified schematic illustrates how neuroinflammatory processes in the aging brain may arise from interactions between CNS cellular and systemic physiological networks. Under homeostatic conditions (upper blue region), coordinated communication among neurons, microglia (MG), astrocytes (AC), pericytes (PC), and components of the neurovascular unit (NVU), including endothelial cells (EC), may support neuronal function and network resilience. Astrocytes and microglia contribute to synaptic regulation and metabolic support, while the neurovascular interface helps maintain controlled exchange between the circulation and the brain. With advancing age (lower red region), systemic alterations across multiple physiological networks may influence neuroimmune signaling within the CNS. Age-associated immune changes, including the accumulation of peripheral senescent immune cells and increased systemic inflammatory mediators (iM), may provide persistent inflammatory input to the brain. At the neurovascular interface, alterations in BBB integrity and endothelial permeability may facilitate enhanced transmission of circulating inflammatory signals and infiltration of peripheral immune cells (pIC). Concurrently, age-related shifts in gut microbiome composition and microbial metabolite production may further modulate systemic immune signaling through the gut–brain–immune axis. These converging systemic inputs may promote glial activation, including reactive astrocytes (rAC) and activated microglia (aMG), thereby amplifying inflammatory signaling within neuronal networks. Such processes can influence synaptic stability, neuronal viability, and circuit function, contributing to increased vulnerability of brain networks during aging. Together, the figure highlights the concept that neuroinflammation may emerge from integrated communication between the brain and peripheral physiological systems rather than solely from CNS-intrinsic mechanisms.

Neuroinflammatory processes in the aging brain may be shaped not only by cellular interactions within the CNS but also by signals originating from peripheral organs and physiological systems. Increasing evidence suggests that communication between the brain and peripheral immune, vascular, and metabolic systems can influence neuroimmune activity across the lifespan (Figure 2). These interactions form a broader physiological network in which systemic changes associated with aging may modulate inflammatory signaling within the CNS (Müller et al., 2025d; Seguin et al., 2023; Zheng et al., 2025).

### 3.1 Peripheral immune aging

Age-related changes in immune function can modify the systemic inflammatory environment and thereby influence neuroimmune signaling (Di Benedetto et al., 2017; Müller and Di Benedetto, 2024; Di Benedetto et al., 2019a). The aging immune system often exhibits features collectively described as immunosenescence, including altered immune cell composition, reduced adaptive immune diversity, and changes in functional responsiveness of both innate and adaptive immune cells. At the same time, aging has been associated with a chronic low-grade inflammatory state frequently referred to as inflammaging.

Elevated levels of circulating pro-inflammatory cytokines, chemokines, and related inflammatory mediators such as IL-1β, IL-6, TNF-α, and CCL2 may provide sustained peripheral input into regulatory pathways, defined here as endothelial–glial–neuronal signaling networks at the neurovascular unit (Ge et al., 2008). These mediators act primarily through endothelial activation (e.g., NF-κB–dependent upregulation of adhesion molecules and altered blood–brain barrier permeability), thereby promoting cytokine and secondary messenger signaling into the CNS (Pribiag and Stellwagen, 2013; Paul et al., 2014). This, in turn, can induce a primed or reactive glial phenotype in microglia and astrocytes, characterized by enhanced sensitivity to secondary inflammatory stimuli and altered cytokine release profiles. Downstream effects include modulation of neuronal excitability and synaptic plasticity, particularly through cytokine-dependent regulation of glutamatergic and GABAergic signaling, which may, over prolonged periods, contribute to subtle but sustained alterations in network connectivity and synaptic homeostasis (Müller and Di Benedetto, 2026; Franceschi et al., 2018; Stellwagen and Malenka, 2006).

Peripheral inflammatory mediators can interact with brain cells through several routes, including receptor-mediated signaling at the neurovascular interface, transport across specialized barrier systems, and indirect modulation of glial activity via endothelial and perivascular cells. Through these mechanisms, systemic inflammatory states may alter how microglia and astrocytes respond to physiological stimuli and environmental stressors. As a result, peripheral immune dynamics can become functionally integrated into neuroinflammatory regulation within the CNS (Figure 2), contributing to long-range coupling between systemic physiology and brain-resident immune activity (Franceschi et al., 2018; Tamatta et al., 2025; Soraci et al., 2024). These systemic inflammatory changes may prime CNS-resident cells toward altered responsiveness, thereby linking peripheral immune aging with local neuroimmune network dynamics.

### 3.2 The neurovascular unit: focus on blood–brain barrier

The BBB, formed primarily by specialized endothelial cells within the neurovascular unit, represents a critical communication interface between systemic physiology and brain tissue that tightly regulates the exchange of molecules and cells between the circulation and the CNS. Beyond its barrier function, the neurovascular unit—including endothelial cells, pericytes, astrocytic endfeet, and associated immune components—acts as an active signaling platform that integrates peripheral and central cues (Kadry et al., 2020; Neyra Chauca et al., 2026).

Through endothelial signaling pathways, transport mechanisms, and interactions with glial cells, the neurovascular interface may actively regulate how systemic inflammatory mediators influence CNS cellular networks. Age-associated alterations in these regulatory processes could therefore modify glial responsiveness, inflammatory signaling, and neuronal communication, potentially amplifying the impact of peripheral inflammatory states on brain function during aging (Iadecola, 2017; Hu et al., 2025).

During aging, structural and functional modifications of the neurovascular interface may alter the transmission of inflammatory signals between the circulation and the brain. Changes in vascular integrity, endothelial signaling, and transport mechanisms could influence the passage of cytokines, metabolic factors, or immune cells into the CNS microenvironment (Figure 2). These processes may amplify local inflammatory signaling or modify the sensitivity of CNS-resident cells to systemic cues. Consequently, the neurovascular interface can function as a key regulatory node linking peripheral immune activity with brain inflammatory dynamics (Neyra Chauca et al., 2026; Iadecola, 2017; Castro and Potente, 2022; Graves and Baker, 2020). Altered neurovascular signaling may therefore amplify inflammatory communication between the periphery and the CNS during aging.

### 3.3 Gut–brain–immune communication

The gut microbiome represents another systemic component that may participate in neuroimmune regulation (Figure 2). The composition and diversity of the gut microbiota can change with age, influenced by diet, lifestyle, medication use, and physiological factors. Microbial communities interact closely with the host immune system and contribute to the production of metabolites capable of influencing inflammatory pathways.

Studies of aging populations have reported reductions in microbial diversity together with shifts in the relative abundance of specific bacterial taxa and alterations in microbial metabolic activity (Claesson et al., 2012; Claesson et al., 2011). These changes may be accompanied by reduced production of beneficial metabolites, including certain short-chain fatty acids, and by impaired intestinal barrier integrity, potentially increasing exposure to microbe-derived inflammatory signals. Age-related shifts in microbiome composition and metabolic output may therefore modify immune signaling at the organismal level (Müller and Di Benedetto, 2025c; Shabbir et al., 2021; O'Riordan et al., 2025).

Microbial metabolites such as short-chain fatty acids, tryptophan derivatives, and other bioactive molecules can interact with immune and neural signaling pathways, potentially affecting glial activity and inflammatory responses. These signals may influence the CNS through several partially interconnected routes, including release of microbial metabolites into the systemic circulation, modulation of peripheral immune signaling pathways, interactions with the neurovascular interface, and vagal nerve-associated communication between the gut and the brain. Through these mechanisms, microbiome-associated signaling may indirectly shape glial activity and neuroinflammatory regulation during aging (Needham et al., 2020; Bostick et al., 2022).

Collectively, these signaling pathways may integrate gut-derived signals into broader neuroimmune networks connecting metabolic, immune, and neural systems. In particular, these metabolites can influence peripheral immune cell differentiation and cytokine production, modulate BBB permeability, and engage host receptors such as G-protein coupled receptors and aryl hydrocarbon receptors that are expressed on immune and neural cells. As a result, variations in gut microbial composition may translate into measurable changes in central immune tone and microglial activation states, thereby contributing to inter-individual differences in neuroinflammatory regulation (Müller and Di Benedetto, 2025c; Shabbir et al., 2021; O'Riordan et al., 2025; Kalyanaraman et al., 2024; Collins et al., 2012; Bano et al., 2024; Silva et al., 2020; Gao et al., 2020). Through these mechanisms, microbiome-associated signals may modulate glial activity and influence inflammatory regulation within aging neural networks.

### 3.4 Integration of systemic and central inflammatory signaling

Together, these observations suggest that neuroinflammatory activity may reflect interactions between the brain and multiple peripheral systems. Immune mediators, vascular interfaces, and microbiome-derived signals may collectively influence how inflammatory responses are initiated and regulated within the CNS (Müller et al., 2025d; Di et al., 2019; Chalmers et al., 2022).

In addition to immune and microbiome-associated pathways, age-related dysfunction in peripheral organs such as the cardiovascular system may further contribute to circulating inflammatory mediators, metabolic alterations, and vascular signaling changes capable of influencing blood–brain barrier integrity, neuronal function, and neuroimmune regulation (Smith et al., 2024). Within this framework, neuroinflammation may be understood as a systems-level phenomenon shaped by continuous bidirectional communication between the brain and the body (Müller et al., 2025d; Smith et al., 2024). Such an integrated perspective highlights the importance of considering multi-system interactions when examining the mechanisms that govern inflammatory regulation and brain aging trajectories.

Importantly, interpretation of systemic neuroimmune interactions remains complicated by substantial methodological heterogeneity across studies. Differences in animal models, microbiome composition, dietary conditions, inflammatory paradigms, and biomarker selection may influence reported outcomes and limit direct comparability between studies. In human investigations, variability in age ranges, comorbidities, and imaging or circulating inflammatory markers may further complicate the identification of consistent neuroinflammatory signatures associated with aging.

Together, these findings suggest that systemic immune, vascular, and microbiome-derived signals may actively shape neuroinflammatory network dynamics by modulating glial activation states and inflammatory communication within the CNS, particularly in the context of aging.

## 4 Neuroinflammation and network vulnerability in brain aging

Alterations in neuroinflammatory signaling may influence the stability and functional organization of neural circuits during aging. Because neuronal and glial cells operate within highly interconnected networks, changes in inflammatory tone can affect processes ranging from synaptic regulation to large-scale circuit dynamics. In this context, neuroinflammation may contribute to differences in how brain networks adapt to physiological stressors and maintain cognitive function over time (Müller et al., 2025d; Zheng et al., 2025).

### 4.1 Synaptic and circuit alterations

Inflammatory signaling pathways are increasingly recognized as potential modulators of synaptic organization and plasticity. Molecular systems that participate in immune communication within the brain may also contribute to mechanisms involved in synaptic remodeling. Among these pathways, complement-related signaling has received particular attention. Experimental studies suggest that complement components can participate in synaptic pruning processes, which under physiological conditions may support circuit refinement and removal of dysfunctional synapses (Stevens et al., 2007). However, altered regulation of these pathways during aging could influence the balance between synaptic maintenance and elimination (Soteros and Sia, 2022; Zhang et al., 2023a).

At physiological levels, these signaling pathways may support adaptive synaptic remodeling by facilitating removal of dysfunctional synaptic elements and modulating activity-dependent plasticity. However, prolonged or excessive inflammatory activation could shift these mechanisms toward maladaptive synaptic elimination, altered neuronal excitability, and impaired circuit flexibility, thereby affecting network stability and learning-related processes (Head et al., 2009; Zhao et al., 2024).

Inflammatory mediators released by glial cells may also affect neuronal excitability and synaptic transmission. Cytokines and other immune-related molecules have been shown in experimental systems to influence long-term potentiation, neurotransmitter release, and other forms of synaptic plasticity. Changes in these signaling pathways could therefore modify the ability of neural circuits to adapt during learning and memory processes. While many of these mechanisms have been characterized primarily in experimental models, they suggest potential pathways through which inflammatory signaling might influence functional properties of brain networks (Prieto and Cotman, 2017; Stellwagen and Malenka, 2006; Villarreal et al., 2002). Persistent activation of immune-related synaptic remodeling pathways may therefore contribute to reduced circuit stability and altered cognitive adaptability.

### 4.2 Cognitive decline and resilience

The functional consequences of neuroinflammation during aging may vary considerably among individuals. Some studies have reported associations between elevated inflammatory markers and measures of cognitive decline, suggesting that cumulative inflammatory exposure—sometimes described as inflammatory load—could influence cognitive trajectories over time (Mar et al., 2015; West et al., 2020). However, these relationships are complex and may be shaped by genetic background, environmental exposures, and systemic physiological conditions (Gonzalez-Bautista et al., 2025; Di et al., 2019; Di Benedetto et al., 2019b; Zhang W. et al., 2025).

Aging populations display substantial heterogeneity in cognitive outcomes, suggesting that neuroinflammatory processes may interact with genetic, metabolic, vascular, and environmental factors that collectively influence neural network resilience and cognitive trajectories (West et al., 2020; Noss et al., 2022; Müller et al., 2026b). Variability in immune regulation, cellular stress responses, and metabolic state may influence how neural networks respond to inflammatory signaling (Müller et al., 2025b; Di et al., 2019). In some cases, compensatory mechanisms within neural circuits may help preserve cognitive performance despite the presence of inflammatory changes (Di Benedetto et al., 2019b; Kanishka and Jha, 2023; Zhang et al., 2026). Understanding how such resilience mechanisms operate remains an important challenge for aging research, particularly with respect to identifying the cellular and network-level adaptations that support preserved function in the face of chronic low-grade inflammation. The functional impact of neuroinflammatory signaling may thus depend on the capacity of neural networks to maintain compensatory and adaptive responses during aging.

### 4.3 Links to neurodegenerative disease

Persistent inflammatory activity in the aging brain has also been proposed as a factor that may influence vulnerability to neurodegenerative disorders. Chronic inflammatory signaling could alter cellular homeostasis, synaptic integrity, and metabolic balance in ways that create conditions permissive for the accumulation of pathological proteins or neuronal dysfunction (Di Benedetto et al., 2017; Müller et al., 2025a). In this sense, aging-related inflammation may interact with disease-specific mechanisms rather than acting as an independent cause of neurodegeneration. Prolonged activation of glial cells, for example, may affect protein clearance pathways such as autophagy and proteasomal degradation, while also influencing BBB integrity and the exchange of immune mediators between the CNS and the periphery (Müller et al., 2025c; Tamatta et al., 2025; Takata et al., 2021; Liu et al., 2022).

Evidence from experimental models and human studies suggests that inflammatory pathways can interact with processes involved in neurodegenerative disorders. In Alzheimer’s disease, amyloid-β accumulation, tau-associated pathology, and synaptic dysfunction may interact closely with neuroinflammatory signaling pathways. Experimental and human studies suggest that amyloid-β aggregates can promote glial activation and inflammatory mediator release, whereas chronic inflammatory signaling may further enhance tau-associated pathology, promote excessive microglia-mediated synaptic elimination, and contribute to reduced neuronal network stability. Through these bidirectional interactions, persistent neuroinflammatory activity may increase the vulnerability of neural circuits and facilitate neurodegenerative progression (Gratuze et al., 2023; Ising et al., 2019; Versele et al., 2022; Sommer et al., 2017; Abdelhamed et al., 2025).

However, the temporal relationship between neuroinflammation and disease pathology remains an area of ongoing investigation, with some studies indicating that immune activation may precede overt protein aggregation, while others suggest it emerges as a response to existing pathology. It is possible that age-associated inflammatory changes initially arise as adaptive responses to cellular stress but may become maladaptive when regulatory mechanisms fail to restore homeostasis, leading to sustained feedback loops between immune activation, neuronal vulnerability, and progressive network dysfunction (Müller et al., 2025a; Zhang et al., 2023b).

A major challenge in the field involves distinguishing inflammatory processes associated with physiological aging from those linked to early or subclinical neurodegenerative pathology. Because many inflammatory pathways may be activated across both conditions, interpretation of neuroinflammatory markers often remains context-dependent. Furthermore, glial phenotypes identified in experimental systems may not fully correspond to cellular states observed in the human brain, highlighting the importance of cautious translation between *in vitro* studies, animal models, and human investigations (Suk, 2026).

Chronic inflammatory signaling may thereby create conditions that facilitate the transition from adaptive aging responses toward neurodegenerative network dysfunction.

### 4.4 Network vulnerability and functional outcomes

Taken together, these findings indicate that neuroinflammation may influence brain aging by modifying the stability and adaptability of neural networks. Changes in inflammatory signaling can affect synaptic regulation, circuit plasticity, and neuronal communication, potentially altering how brain networks respond to physiological and environmental challenges. From this perspective, neuroinflammation may not only reflect underlying pathology but may also participate in shaping the balance between resilience and vulnerability that characterizes cognitive aging (Müller and Di Benedetto, 2026). In this context, neuroinflammatory signaling may influence cognitive aging by modulating the balance between adaptive synaptic remodeling and maladaptive circuit destabilization.

## 5 Emerging approaches to study neuroinflammatory networks

Advances in experimental technologies and analytical frameworks are increasingly enabling the study of neuroinflammation as a multiscale and dynamic process. Traditional approaches that focused on individual cell types or isolated signaling pathways are gradually being complemented by methodologies capable of capturing cellular diversity, spatial organization, and systemic interactions within neuroimmune networks. Such approaches may help clarify how inflammatory processes evolve across different stages of brain aging and how cellular and systemic signals become integrated within the CNS (Zou et al., 2025; Sun et al., 2024; Adewale et al., 2021; Vitorino, 2024).

### 5.1 Multi-omics and single-cell approaches

Recent developments in multi-omics technologies have expanded the capacity to characterize molecular and cellular states associated with neuroinflammation. Techniques such as single-cell transcriptomics, epigenomic profiling, and spatially resolved molecular analyses can provide detailed insights into the diversity of glial cell populations and their functional states. Studies using these approaches have suggested that microglia and astrocytes may exist in multiple transcriptional and metabolic states that vary across brain regions, environmental conditions, and stages of aging (Sun et al., 2024; Ren et al., 2025; Wang et al., 2023; Kocar et al., 2026; Voicu et al., 2025; Ali et al., 2025; Tsai et al., 2025; Ramamurthy et al., 2022).

For example, single-cell and spatial transcriptomic studies have identified region-specific microglial and astrocytic states associated with aging and neurodegenerative conditions, revealing substantial heterogeneity in neuroimmune responses across different brain regions and physiological contexts. Such approaches may also help identify molecular programs associated with either adaptive or maladaptive inflammatory trajectories during aging (Tsai et al., 2025).

By resolving cellular heterogeneity, multi-omics strategies may help identify molecular signatures associated with distinct inflammatory responses or adaptive cellular programs. In addition, longitudinal or cross-sectional datasets can potentially reveal trajectories of cellular change that occur during aging. Integrating transcriptomic, proteomic, and metabolomic data may therefore contribute to a more comprehensive understanding of how neuroinflammatory signaling evolves over time within cellular networks (Nourazarain and Vaziri, 2025; Li et al., 2026).

### 5.2 Neuroimaging of brain inflammation

Non-invasive imaging techniques have also become increasingly important for investigating neuroinflammatory processes in the human brain. Positron emission tomography (PET) imaging using radioligands that bind to proteins associated with glial activation has been widely used to estimate neuroimmune activity *in vivo*. Although these signals may not be entirely cell-type specific, PET-based approaches can provide insights into regional patterns of neuroinflammatory activity and their potential relationships with aging, cognitive function, or disease processes (Suridjan et al., 2014; Adewale et al., 2021). For instance, PET imaging using translocator protein (TSPO)-associated radioligands has been widely applied to examine regional glial activation patterns in aging and neurodegenerative disorders (Salerno et al., 2024).

Magnetic resonance imaging (MRI) methods have been explored to assess vascular integrity, tissue microstructure, and inflammatory-associated alterations in neural connectivity. In parallel, advanced MRI approaches have also been explored as indirect markers of neuroinflammatory processes. Sophisticated MRI techniques may capture changes in tissue microstructure, vascular integrity, or metabolic activity that could reflect inflammatory alterations within the brain (Zhang et al., 2026; Chen et al., 2026). When combined with other biomarker data, such imaging approaches may help characterize spatial and temporal patterns of neuroinflammatory activity across the aging brain (Adewale et al., 2021; Talukdar et al., 2023; Vinci-Booher et al., 2025).

### 5.3 Systems and computational approaches

Because neuroinflammatory processes involve complex interactions among multiple cell types and physiological systems, computational and systems-level approaches are increasingly being used to analyze large-scale datasets and model underlying network dynamics. In particular, network modeling strategies can help identify key regulatory nodes within neuroimmune signaling pathways and elucidate how perturbations in one component of the system may propagate to influence broader network behavior (Kempuraj et al., 2026; Müller et al., 2025a; Seguin et al., 2023; Kocar et al., 2026; Bauer and Thiele, 2018; Subramanian et al., 2015; Mheich et al., 2020).

Multilayered network analyses may offer a powerful framework for capturing neuroimmune interactions by representing the brain, immune system, and other physiological systems as interconnected, multi-scale networks rather than isolated entities. Within this framework, distinct biological layers—such as gene regulatory networks, intracellular signaling cascades, cell–cell communication networks, and systemic immune interactions—can be modeled as separate but interdependent layers within a unified architecture. This structure makes it possible to trace how changes in one layer, such as microglial activation or peripheral cytokine signaling, cascade across levels to affect neuronal activity, synaptic remodeling, and ultimately behavior (Müller et al., 2025a; Müller et al., 2025c; Zhou and Jiang, 2025).

Although emerging multi-omics and computational approaches provide unprecedented resolution for studying neuroimmune networks, integration of datasets across platforms and species remains challenging. Differences in analytical pipelines, cell classification strategies, and spatial resolution may contribute to discrepancies between studies. Future efforts aimed at harmonizing methodological approaches and combining longitudinal human datasets with experimental models may therefore be important for improving reproducibility and interpretation within the field (Ren et al., 2025; Voicu et al., 2025; Sibilio et al., 2025).

Importantly, multilayer models allow for the integration of heterogeneous data types, including transcriptomic, proteomic, imaging, and clinical data, thereby enabling the detection of cross-layer hubs and feedback loops that are not readily identifiable in single-layer analyses (Kempuraj et al., 2026; Sibilio et al., 2025; Lee et al., 2019; Edahiro et al., 2025; Gong et al., 2025). By uncovering emergent properties of neuroimmune crosstalk—such as state-dependent shifts between neuroprotective and neurotoxic immune responses—this approach moves beyond reductionist perspectives toward a more holistic, systems-level understanding of brain–immune dynamics in health and disease (Castellani et al., 2023).

Complementing these approaches, integrative data-driven frameworks that combine molecular, cellular, imaging, and clinical information provide additional opportunities to study neuroinflammatory processes across multiple biological scales. Machine learning and related computational techniques can further support the identification of complex patterns and associations that may not be apparent using traditional analytical methods (Jamialahmadi et al., 2024; Hanna et al., 2025; Neumann, 2025).

Network-based computational analyses integrating transcriptomic, imaging, and clinical datasets have also begun to identify coordinated inflammatory signaling modules associated with cognitive decline and neural vulnerability. Such integrative frameworks may help clarify how cellular and systemic inflammatory processes become functionally interconnected across different stages of brain aging (Bhat et al., 2026; Li et al., 2025). Together, these approaches hold promise for developing more comprehensive and predictive models of neuroinflammatory regulation during aging.

### 5.4 Toward multiscale integration

Collectively, these emerging methodologies are promoting a more integrative perspective on neuroinflammation that spans molecular, cellular, and systems levels of organization. By combining high-resolution molecular profiling with *in vivo* imaging and computational modeling, it is becoming possible to reconstruct the complex, dynamic multilayered networks that link immune signaling to brain structure and function. Within this multiscale framework, neuroinflammation can be understood not as an isolated process, but as an emergent property of interacting biological systems operating across different spatial and temporal scales. Such an integrated perspective is likely to be essential for elucidating how inflammatory processes shape trajectories of brain aging and for identifying robust, system-level targets for interventions aimed at preserving neural health and resilience. Finaly, such multiscale approaches may help distinguish physiological neuroimmune adaptations from dysregulated inflammatory states associated with neural vulnerability during aging.

## 6 Future directions and conclusions

Current evidence suggests that neuroinflammatory processes may act as a central hub within the biological networks that shape brain aging. Rather than representing a single pathway or isolated mechanism, neuroinflammation appears to intersect with multiple physiological domains, including immune regulation, metabolic signaling, vascular function, and microbial interactions. Through these connections, inflammatory signaling may integrate diverse systemic inputs and translate them into cellular responses within neural circuits. From this perspective, neuroinflammation can be viewed as a dynamic regulatory interface through which aging-related changes across the organism may influence brain structure and function.

Despite substantial progress in characterizing components of neuroimmune signaling, important questions remain regarding the temporal dynamics and causal relationships underlying these processes in humans. Longitudinal studies that combine molecular biomarkers, neuroimaging measures, and cognitive assessments may therefore be essential for understanding how neuroinflammatory activity evolves over the course of aging. Such approaches could help clarify whether specific inflammatory patterns precede, accompany, or follow functional changes in neural networks and cognitive performance.

Future research may also benefit from the development of systems-level models that integrate data across biological scales. Computational frameworks capable of incorporating molecular, cellular, physiological, cognitive, and behavioral information may provide new opportunities to explore how interactions among different components of neuroimmune networks influence aging trajectories. These integrative approaches could help identify regulatory nodes or signaling pathways that disproportionately influence network behavior and may therefore represent potential targets for intervention.

Although multi-omics and imaging technologies provide increasingly detailed characterization of neuroimmune states, important limitations remain, including variability in biomarker interpretation, limited temporal resolution, and challenges associated with integrating datasets across experimental models and human populations. Addressing these limitations may be important for the development of more precise and individualized strategies aimed at preserving brain health during aging.

An additional priority involves identifying modifiable factors that influence neuroinflammatory regulation. Future studies may particularly benefit from examining how environmental exposures, metabolic health, dietary patterns, physical activity, sleep quality, and microbiome-associated factors interact with neuroimmune signaling across aging trajectories. Because these variables can differ substantially between individuals, integrative longitudinal approaches may help identify personalized neuroinflammatory profiles associated with either resilience or vulnerability to cognitive decline. Lifestyle, environmental exposures, metabolic health, and microbial composition have all been proposed as variables that may interact with immune signaling during aging. Understanding how such factors affect neuroimmune networks could inform strategies aimed at reducing detrimental inflammatory activity while preserving physiological immune functions.

Together, these perspectives support a view of neuroinflammation as an important integrative component of brain aging biology. Continued efforts to examine neuroinflammatory processes within multiscale physiological networks may help clarify the mechanisms that underlie cognitive decline as well as resilience in aging populations. Ultimately, insights derived from these approaches could contribute to the development of more precise strategies for maintaining brain health and functional independence throughout the lifespan.
